# Composition and Antioxidant Status of Human Milk of Women Living in Bydgoszcz (Poland)

**DOI:** 10.3390/nu16193396

**Published:** 2024-10-06

**Authors:** Agnieszka Dombrowska-Pali, Agnieszka Chrustek, Dorota Olszewska-Słonina, Maciej W. Socha

**Affiliations:** 1 Department of Perinatology, Gynecology and Gynecologic Oncology, Faculty of Health Sciences, Collegium Medicum in Bydgoszcz, Nicolaus Copernicus University, Łukasiewicza 1, 85-821 Bydgoszcz, Poland; 2 Department of Pathobiochemistry and Clinical Chemistry, Faculty of Pharmacy, Collegium Medicum in Bydgoszcz, Nicolaus Copernicus University, M. Curie-Skłodowskiej 9, 85-094 Bydgoszcz, Poland; 3 Department of Obstetrics and Gynecology, St. Adalberts’s Hospital in Gdańsk, Copernicus Healthcare Entity LLC, Jana Pawła II 50, 80-462 Gdańsk, Poland

**Keywords:** human milk, breastfeeding, antioxidation, maternal factors

## Abstract

Objectives: The aim of this study was to compare cortisol concentrations, nutritional composition, and the antioxidant status of human milk of women living in Bydgoszcz (Poland), taking into account maternal factors (fertility, area of residence, economic activity, and breastfeeding period). Methods: The basic composition of human milk was evaluated using the MIRIS HMA^TM^ analyzer. The level of cortisol was determined by the enzyme-linked immunosorbent method. In order to determine the antioxidant activity, the DPPH radical method was used. Results: It was observed that the concentration of cortisol in human milk in the group of women living in the city center was higher compared to the milk of women living on the outskirts of the city. In the group of women breastfeeding from 3 to 5 weeks after childbirth, the concentration of cortisol in milk was higher compared to the group of women breastfeeding less than 12 months of age and compared to the group of women lactating over 12 months of age. The antioxidant status of human milk was highest in the group of professionally active women and in the group of breastfeeding women from 3 to 5 weeks after childbirth. The basic composition and the caloric value of human milk differed statistically significantly in the study groups. Conclusions: Based on this study, it can be concluded that the composition and antioxidant status of human milk depends on maternal factors (fertility, professional activity, area of residence, and breastfeeding period). Higher cortisol concentrations in breast milk are probably determined by the area of residence (city center and associated higher noise/sound and stress levels) and lactation period (hormonal imbalance, fatigue, and postpartum period). Milk from economically active women shows greater protection against reactive oxygen species compared to milk from inactive women, protecting against the occurrence of diseases of civilization. Milk from breastfeeding women over 12 months of age also shows protection against reactive oxygen species, despite the fact that the highest level of antioxidant status of human milk occurs in the initial period of lactation.

## 1. Introduction

Human milk is a composition of nutrients and bioactive components that are quantitatively and qualitatively tailored to meet the needs of the developing child. Human milk is viewed not only as a nutritional substance but also as a functional food [1,2,3,4].

Breastfeeding, or feeding with expressed human milk, due to its immediate and long-term health benefits for both the child and the mother, is referred to as the “gold standard of newborn and infant nutrition”. The World Health Organization (WHO) recommends exclusive breastfeeding for the first 6 months of a child’s life. In the same recommendations, WHO encourages continuing breastfeeding until the child’s second year of life and beyond, while simultaneously providing complementary foods. Furthermore, WHO states that early initiation of breastfeeding—within the first hour after the child’s birth—protects the infant from infections, while simultaneously reducing mortality rates. It is also important to note that milk from mothers of children aged 6–23 months remains a significant source of energy and nutrients. Human milk can cover half or more of the energy needs of a child aged 6 to 12 months and one-third of the energy needs of a child aged 12 to 24 months [1].

The composition of human milk and its antioxidant status varies with the duration of lactation, the duration of a single feed, the time of day, perinatal factors, maternal factors, and environmental factors. This variability has a beneficial effect on the health and survival of infants [5]. Recent studies indicate that over the course of a year, breastfeeding can reduce infant mortality by over eight hundred thousand and maternal mortality by about two hundred thousand [4].

Nutrients in human milk come from three sources: some are synthesized in lactocytes, some are derived from the mother’s diet, and others are mobilized from maternal stores [5]. Human milk can be defined as a bioactive fluid that changes its composition from colostrum to transitional milk and then to mature milk, depending on factors such as pregnancy duration, maternal age, BMI, medication use, dietary habits, and the geographic location of the nursing mother [6,7,8,9,10,11]. A notable example of this is a study conducted in Davis, California, which examined the relationship between maternal characteristics and the macronutrient composition of human milk. It was shown that four months postpartum, macronutrient concentrations in human milk were associated with one or more of the following factors: maternal BMI, protein intake, number of births, return of menstruation, and breastfeeding frequency. The study also revealed that mothers who produce larger amounts of milk tend to have lower concentrations of fat and protein in their milk but higher concentrations of lactose [12].

Given the importance of understanding the composition of human milk as a tool for managing infant feeding, the aim of this study was to assess the composition, caloric content, cortisol levels, and antioxidant status of human milk from women residing in Bydgoszcz (Poland) and to determine the relationship with factors such as lactation stage, parity, area of residence, way of delivery, professional activity, BMI of the breastfeeding woman, and the presence of diseases during pregnancy or lactation. It should be emphasized that, to date, there have been few studies evaluating human milk in this part of Europe [13].

## 2. Materials and Methods

### 2.1. Study Group

This study was conducted in a group of 183 women living in the city of Bydgoszcz (Poland). Women after physiological childbirth and cesarean section staying at the Obstetrics Clinic of Women’s Diseases and Gynecologic Oncology of the Dr. J. Biziel University Hospital No. 2 in Bydgoszcz qualified for the research project, as well as patients after childbirth in other medical facilities who reported their willingness to participate in the research via social media.

The research was approved by the Bioethics Committee of the Nicolaus Copernicus University in Toruń at the Ludwik Rydygier Collegium Medicum in Bydgoszcz (consent nos. KB437/2018 and KB121/2019). All women participating in this study read information about it, completed the questionnaire, and expressed their opinions. The questionnaire design consisted of 58 questions. The first part of the questionnaire included questions about the age of the women, residence, education, civil status, and professional activity. The next questions in the questionnaire concerned the data, i.e., weight and height of the breastfeeding women, duration of pregnancy, fertility (primiparous/multiparous), diseases that occurred during pregnancy and breastfeeding, and how the pregnancy ended (vaginal delivery/cesarean section).

**Figure 1:**
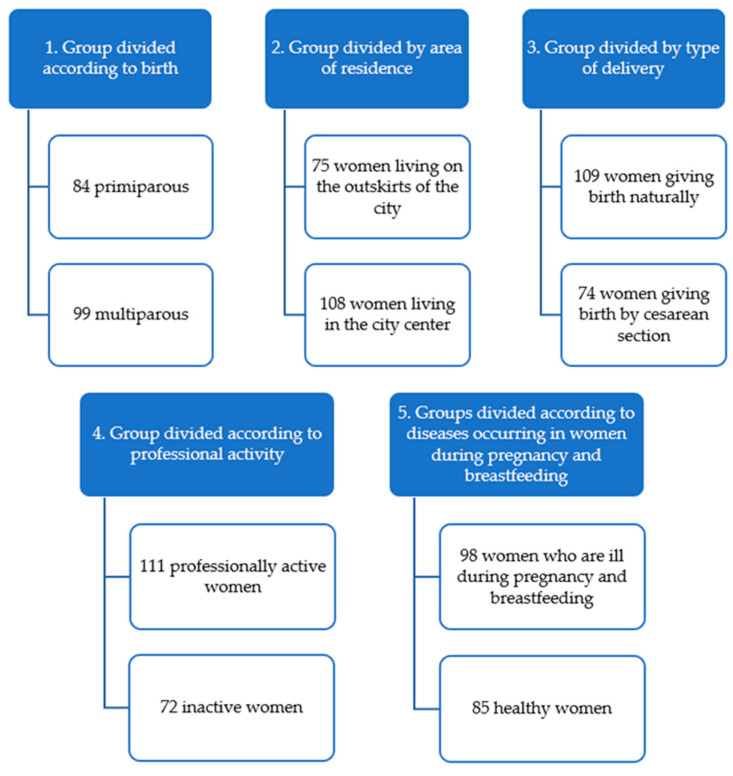

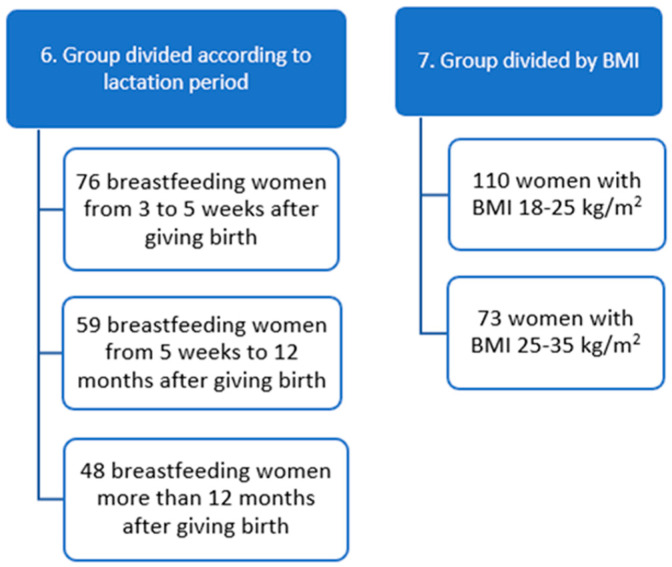
Scheme inclusion and exclusion criteria for study groups (*n* = 183).

In the entire study group (*n* = 183), there were 84 primiparous and 99 multiparous women (Figure 1). Another variable is the area of residence. Women living on the outskirts of the city (*n* = 75) were selected for living in single-family houses or small, single blocks of flats near forests, parks, and far from frequently used roads. The second group of women (*n* = 108) consisted of study participants living in districts with high and medium population density, living in blocks of flats on frequently used streets. This study excluded women after surgical delivery (forceps, vacuum). However, out of 183 women, 109 women gave birth naturally and 74 women gave birth by cesarean section. Another variable is professional activity: 111 professionally active women and 72 professionally inactive women were distinguished. The study group included 85 healthy women and 98 women who were ill during pregnancy and breastfeeding. The comorbidities during pregnancy and lactation were mainly such diseases as thyroid diseases, gestational diabetes, and hypertension. The patients were under strict medical supervision and followed the recommendations. Patients with hypertension and thyroid diseases were treated with pharmacological agents, while the group of patients with gestational diabetes used diet therapy. The study group was also divided depending on the lactation period, and the following were distinguished: 76 breastfeeding women from 3 to 5 weeks after giving birth, 59 breastfeeding women from 5 weeks to 12 months after giving birth, and 48 breastfeeding women more than 12 months after giving birth. The last variable studied was the BMI of breastfeeding women. The group included 110 women with a BMI of 18–25 kg/m^2^ and 73 women with a BMI of 25–35 kg/m^2^.

Groups of breastfeeding women were also analyzed in terms of age, diet, or supplementation. However, the division into groups did not show significant statistical differences. Therefore, these groups are not included in the publication.

### 2.2. Materials

The material for this study was human milk from breastfeeding women. Human milk came from a daily collection (40 mL). During the day, the studied group of women expressed milk by hand in four time slots: 06:00–12:00, 12:00–18:00, 18:00–24:00, and 24:00–6:00. The amount of manually expressed milk in each time interval was 10 mL, with 5 mL before the baby latching on and 5 mL after the end of the feeding. Each portion of milk was poured into one collective bottle. In order to analyze the cortisol concentration in human milk, the study participants expressed a single portion of milk (20 mL) on a fixed day at about 8.30 a.m. The material was delivered within 24 h of collection, portioned into 2 mL Eppendorf tubes (MedLab, Raszyn, Poland), and frozen at −20 °C, followed by −80 °C. The material was stored under these conditions until the analysis of the parameters described below, but no longer than 6 months.

#### 2.2.1. Determination of the Basic Composition of Human Milk

Determination of fat content (mg/100 mL), total protein (mg/100 mL), carbohydrates (mg/100 mL), dry matter (mg/100 mL), and energy value (kcal/100 mL) was carried out. Determination of these parameters was performed in human milk samples using the MIRIS HMA^TM^ (MIRIS AB, Uppsala, Sweden) in accordance with the manufacturer’s procedure (HMA).

The quantitative determination of fat, protein, and carbohydrate content was carried out according to Beer’s law, which states that absorbance is directly proportional to the concentration and path length of the solution through which the radiation passes. The Miris HMA™ software (2022) processes the measurement data through internal calibration. The detection limits (LOD, limit of detection) were as follows: fat content −0.11 g/100 mL, nutritional protein −0.25 g/100 mL, total protein −0.20 g/100 mL, and carbohydrates −0.35 g/100 mL.

Before analysis, human milk samples were heated to 40 °C in a thermostatic bath (BIONOVO, Warsaw, Poland) and then homogenized using the MIRIS Sonicator (1.5 s/mL). Each sample was analyzed in triplicate.

#### 2.2.2. Determination of Cortisol Concentration in Human Milk

The level of cortisol in human milk was determined by the immunoenzymatic method and a commercial test (CORTISOL saliva ELISA DiaMetra, Perugia, Italy). In this method, the combination of cortisol is carried out from the tested human milk reader and the antigen cortisol 2 with horseradish peroxidase (conjugate) with a limited range of anti-cortisol devices coated on a 96-well microplate. To conduct this method, 25 µL (microliter) of human milk was incubated for one hour at 37 °C. For the control, the milk was switched with 200 µL of diluted conjugate. The experiment was performed in duplicate for the purpose of significance. After an hour, the contents of the wells were removed from the microplate and washed three times with 300 µL of previously prepared phosphate buffer. An amount of 100 µL of TMB substrate (3,3′,5,5′-tetramethylbenzidine–3,3′,5,5′-tetramethylbenzidine) was added to each well of the microplate. After 15 slides, the staining was stopped by adding 100 µL of sulfuric acid. The color intensity is an executive device for cortisol in the sample. The absorption of the colored product was measured at a wavelength of 450 nm using a plate reader (Multiskan Go, Thermo Scientific, Waltham, MA, USA). The concentration of cortisol was calculated from the calibration curve (y = 1.66825 + ((0.18995−1.66825)/(1 + (x/5.02629)^−0.832414^)); R^2^ = 0.987). Detection limit: 0.12 ng/mL.

#### 2.2.3. Analysis of the Antioxidant Status of Human Milk

Before the determination, the samples of human milk were gradually thawed in the refrigerator and then centrifuged for 5 min at 10,000× *g* at 4 °C. The defatted supernatant was extracted into new test tubes, which were used for the analysis. Determination of the antioxidant activity of human milk was carried out using the DPPH• (2,2-diphenyl-1-picrylhydrazyl) radical method. To determine the antioxidant activity of human milk, the method of Atanassova et al. (2011) was used with slight modifications [14].

##### Determination in Human Milk Samples

A 100 µM DPPH• solution (Sigma Aldrich, St. Louis, MO, USA) was prepared by dissolving 4 mg of the standard substance in 100 mL of methanol (POCH, Gliwice, Poland). Eppendorf tubes were filled with 1 mL of the prepared DPPH• solution. Next, 250 µL of breast milk was added, vortexed, and then incubated for 60 min in the dark at room temperature. Before reading, the samples were centrifuged for 2 min at 1500× *g* (room temperature). Absorbance was measured at 517 nm against a reference sample, which was methanol. The control sample was a 100 µM methanol DPPH• solution, the absorbance of which was measured at the beginning and end of the experiment.

Solutions for obtaining the calibration curve were prepared in an analogous manner, replacing 250 µL of breast milk with Trolox solutions (TE, (+ −)-hydroxy−2,5,7,8-tetramethylchroman-2-carboxylic acid, Sigma Aldrich, St. Louis, MO, USA) at concentrations of 10–80 mg/L. The standard curve shows the dependence of the Trolox absorbance value on its concentration (y = −0.0101x + 0.8161, R^2^ = 0.9781). The results were expressed in Trolox equivalent (µmol TE/L, mg TE/100 mL). Additionally, the percentage capacity of breast milk to reduce the DPPH• radical was calculated as follows:

$$\%\;inhibition=(\frac{A-Ab}{A})\times 100$$

A—control absorbance.

Ab—average absorbance value of the tested human milk.

Detection limit: 0.1 μg TE/mL.

##### Determination of the Ability of Human Milk to Reduce Fe (III) Ions

The FRAP method (ability to reduce iron ions) according to Benzie and Strain (1999) was used in the experiment with modifications [15].

Before the experiment, the following were prepared: 300 mM of acetate buffer, pH 3.6, and 10 mM TPTZ in 40 mM HCl and 20 mM FeCl_3_·6H_2_O.

The first stage of the determination was to prepare the FRAP reagent by combining the above-mentioned solutions in a ratio of 10:1:1. A blank test was prepared, which was the FRAP reagent, 100 μL of human milk samples, and standard solutions for the determination of the calibration curve. The next step was to subject 50 μL of sample and 1.5 mL of FRAP reagent to vortexing followed by immediate sample absorbance reading (at 0 min) at 593 nm and incubation in a water bath at 37 °C for 4 min. After the incubation period. The samples were thoroughly mixed and the absorbance was measured again at the same wavelength.

A total of 6 solutions of ascorbic acid (Chempur, Piekary Śląskie Poland) were used to determine the calibration curve with concentrations of 100–1000 μM. FRAP concentrations were expressed as the equivalent of ascorbic acid (μM) calculated from the standard curve (y = 0.0006x − 0.0392, R^2^ = 0.9916). The FRAP value was calculated from the following formula:

$$FRAP=\frac{sample\;absorbance\;change\;from\;0\;min\;to\;4\;min}{standard\;absorbance\;change\;from\;0\;\min\;to\;4\;min}\times standard\;concentration\times 2\;[\mu M]$$

Ascorbic acid has a constant stoichiometric coefficient of 2.0 in the FRAP test.

Detection limit: 2 μM.

### 2.3. Statistical Analysis

In order to carry out the statistical analysis, the Statistica 13.1 software package from StatSoft^®^ (Kraków, Poland) was used. The normality of the schedule was verified by the Shapiro–Wolf test. No normality of the distribution of the analyzed quantitative variables was found. A nonparametric Mann–Whitney U test was used to assess statistical significance in two groups of independent variables. In order to compare many groups of independent variables, the Kruskal–Wallis test was used. The variability in the parameters was presented as the median of the lower and upper quartiles (Q25–Q75).

In order to analyze many factors per variable in the studied groups, multiple linear regression was used. The variability in the parameters was presented as the regression coefficient, standard error of the regression coefficient, and the statistical significance level of *p* < 0.005.

To assess the correlation between the parameters studied, the Spearman correlation test was used and the results at the level of *p* < 0.05 were considered statistically significant. The strength of the relationship between the parameters (values of the correlation coefficient) was interpreted according to J. Guilford. as follows:

- |rs| < 0.2—no linear relationship.
- 0.2 ≤ |rs| < 0.4—weak dependency.
- 0.4 ≤ |rs| < 0.7—moderate dependence.
- 0.7 ≤ |rs| < 0.9—strong dependency.
- |rs| ≥ 0.9—a very strong dependency.

The correlations that occur are represented by a correlation matrix. To describe the results, correlations with moderate, strong, and very strong dependence were selected because too many correlations with weak dependence were found.

## 3. Results

The general characteristics of the participants are presented in Table 1. The majority of breastfeeding women are multiparous (54%), gave birth naturally (59%), and live in the city center (60%). A total of 60% of the participants were professionally active, 80% did not take stimulants, and 79% used supplements.

### 3.1. Characteristics of Study Groups

Table 2 presents the study groups by age, BMI, and HBD (hebdomas graviditatis). The BMI of women breastfeeding for 3 to 5 weeks was 7.9% higher than the group of women breastfeeding for 5 weeks to 12 months, and 12.75% higher than the group of women breastfeeding for more than 12 months. The HBD in the group of women breastfeeding from 5 weeks to 12 months and over 12 months after delivery was 14.29% higher compared to the group of women breastfeeding from 3 weeks to 5 weeks after delivery.

### 3.2. Analysis of Cortisol Concentration, Basic Composition, and Antioxidant Status of Human Milk

Statistically significant differences were observed in the basic composition, antioxidant status of breast milk, and cortisol concentration in milk between the study groups (Table 3).

A 31.24% higher cortisol concentration was shown in the milk of women living in the city center compared to the milk of women living on the outskirts of the city. In the group of women breastfeeding from 3 weeks to 5 weeks after delivery, a higher cortisol concentration was observed in breast milk compared to the group of women breastfeeding from 5 weeks to 12 months (by 53.78%) and to the group of women breastfeeding more than 12 months after delivery (35.75%).

The antioxidant status of breast milk was higher by 9.71% in the group of professionally active women compared to the group of inactive women. In addition, a lower level of antioxidant status of the milk of women breastfeeding from 5 weeks to 12 months was observed by 19.82%, as well as milk of women breastfeeding for more than 12 months by 13.20% compared to the antioxidant status of milk of women breastfeeding from 3 weeks to 5 weeks. Additionally, a higher reduction in the DPPH radical by 5% was observed in milk samples of women with a BMI of 25–35 kg/m^2^ compared to the control group. The basic composition of human milk differs depending on the groups studied. The fat content in milk was higher by 39.47% in the group of multiparous women compared to the group of primiparous women, while it was lower by 42.19% in the milk of women giving birth by cesarean section compared to the milk of women giving birth naturally, and also by 40.58% in the milk of women sick during pregnancy and breastfeeding compared to the milk of healthy women. The concentration of fats in human milk increased with the length/time of lactation. Additionally, a 68% higher fat content was observed in the milk of women living on the outskirts of the city compared to the control group.

A higher concentration of total protein was shown in the milk of professionally active women (by 8.34%) compared to the milk of professionally inactive women. The milk of women breastfeeding from 3 weeks to 5 weeks after delivery also showed a higher concentration of total protein compared to the milk of women breastfeeding from 5 weeks to 12 months after delivery. The dry matter content of breast milk was higher in the group of women giving birth naturally (by 4.70%) compared to the group of women giving birth by cesarean section and in the group of professionally active women (by 5.47%) compared to the group of professionally inactive women, as well as in the group of women breastfeeding for over 12 months (by 7.20%) compared to the group of women breastfeeding from 3 weeks to 5 weeks after pregnancy.

A lower caloric content of breast milk was observed in the group of women breastfeeding from 5 weeks to 12 months by 12.99% compared to the group of women breastfeeding for over 12 months, and by 9.46% compared to the group of women breastfeeding from 3 weeks to 5 weeks after delivery. Additionally, a 52% higher fat level was observed in the milk of women with a BMI of 18–25 kg/m^2^ compared to the control group.

### 3.3. Correlations

Many correlations were observed between the analyzed parameters in the study groups (Supplementary Materials Tables S1−S14).

**Figure 2:**
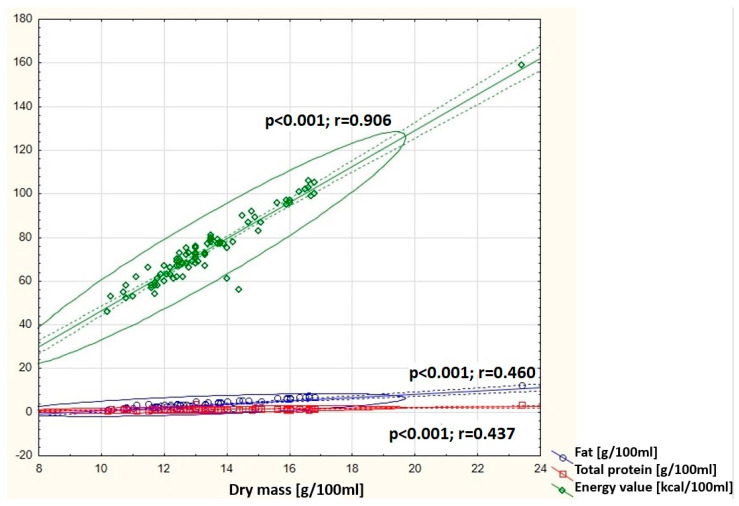
Graphic representation of the correlation between dry mass concentration in milk samples from primiparous women and fat (y = −6.8952 + 0.7595x), energy value (y = −36.3205 + 8.2755x), and total protein (y = −0.2373 + 0.1142x) concentrations. Circle—prediction elipse (95%), dotted line—confidence interval limit, solid line—regression line, dots—data point.

**Figure 3:**
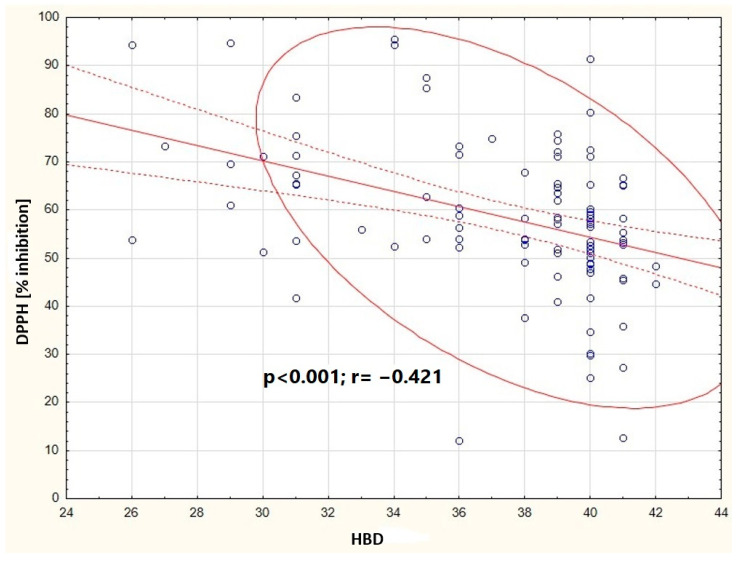
Graphic representation of the correlation between DPPH in milk samples from the multiparous women and the HBD (y = 117.964 − 1.5919x). Circle—prediction elipse (95%), dotted line—confidence interval limit, solid line—regression line, dots—data point.

It was shown that in the group of primiparous women, the concentration of dry matter in human milk positively correlates with the fat content (*p* < 0.001), total protein (*p* < 0.001), and caloric value of human milk (*p* < 0.001) (Figure 2). In the group of multiparous women, a negative correlation was observed between the antioxidant status of human milk (DPPH) and HBD (*p* < 0.001) (Figure 3).

**Figure 4:**
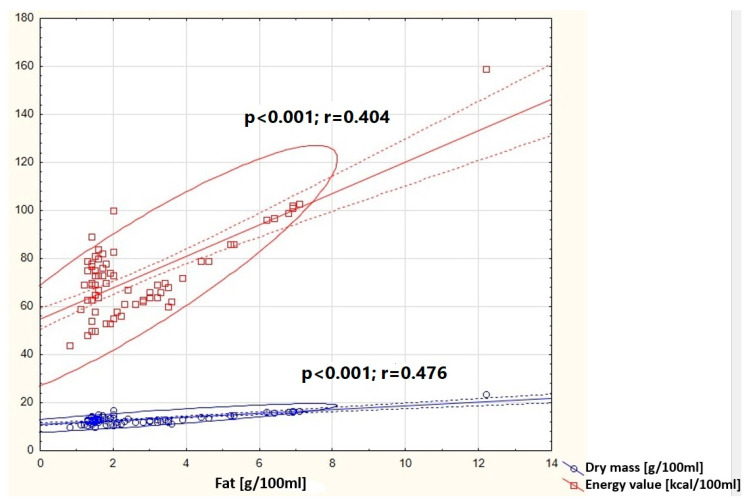
Graphic representation of the correlation between fat concentration in milk samples from women giving birth naturally and the dry mass (y = 11.0761 + 0.7612x) and energy value (y = 54.7909 + 6.5314x). Circle—prediction elipse (95%), dotted line—confidence interval limit, solid line—regression line, dots—data point.

In the group of women giving birth naturally, positive correlations were shown between the concentration of fat in milk and the dry matter content (*p* < 0.001) and the caloric value of milk (*p* < 0.001) (Figure 4).

**Figure 5:**
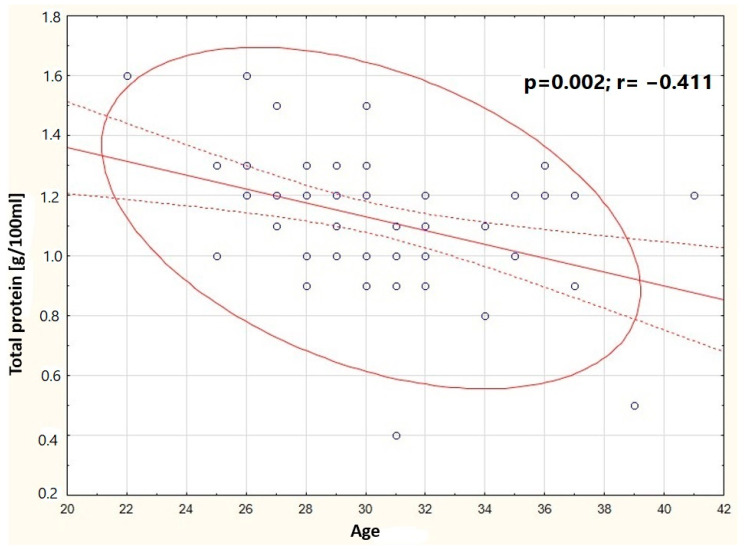
Graphic representation of the correlation between total protein concentration in milk samples from women breastfeeding from 5 weeks to 12 months and the mother’s age (y = 1.8221 − 0.0231x). Circle—prediction elipse (95%), dotted line—confidence interval limit, solid line—regression line, dots—data point.

**Figure 6:**
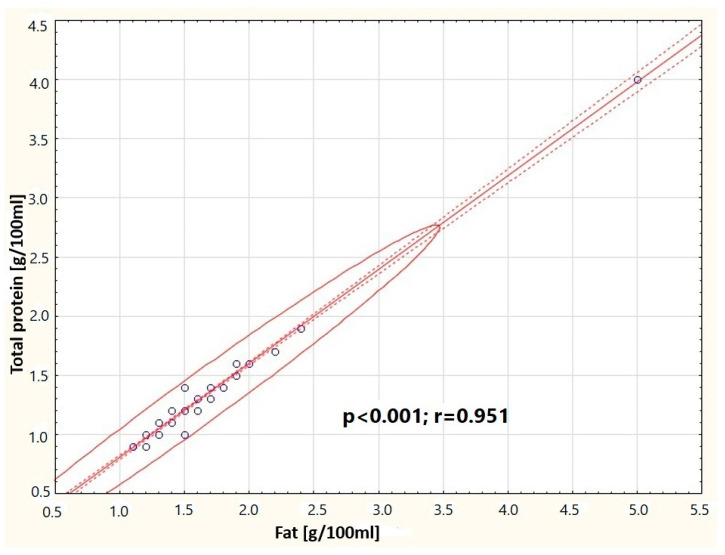
Graphic representation of the correlation between total protein concentration in transitional milk samples and fat concentration (y = 0.0149 + 0.7926x). Circle—prediction elipse (95%), dotted line—confidence interval limit, solid line—regression line, dots—data point.

**Figure 7:**
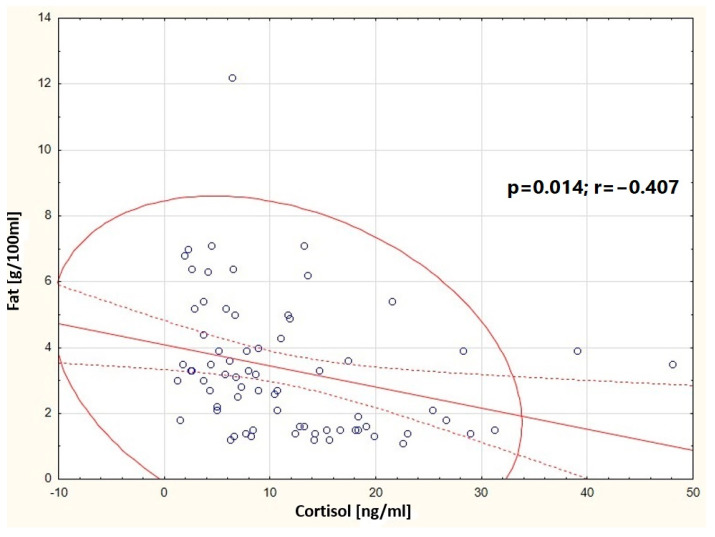
Graphic representation of the correlation between cortisol concentration in milk samples from professionally inactive women and fat concentration (y = 4.0735 − 0.0641x). Circle—prediction elipse (95%), dotted line—confidence interval limit, solid line—regression line, dots—data point.

**Figure 8:**
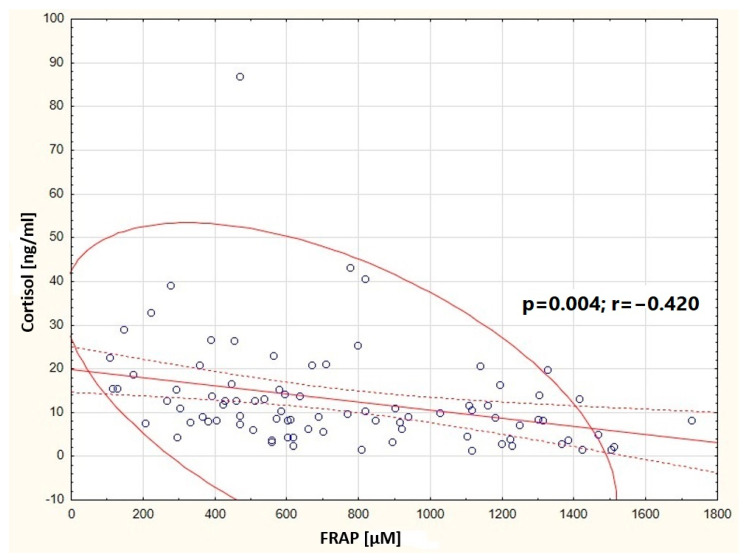
Graphic representation of the correlation between cortisol concentration in milk samples from healthy women and FRAP (y = 19.8395 − 0.0093x). Circle—prediction elipse (95%), dotted line—confidence interval limit, solid line—regression line, dots—data point.

It was observed that in the group of women breastfeeding from 5 weeks to 12 months, the concentration of cortisol in milk negatively correlates with the concentration of fats in milk (*p* = 0.014) (Figure 5). In the group of women breastfeeding from 3 to 5 weeks, a positive correlation was found between the content of total protein and the content of fats in milk (*p* < 0.001) (Figure 6). A negative correlation was found between the concentration of cortisol in milk and the content of fats (*p* = 0.014) in the group of professionally inactive women (Figure 7). Additionally, it was noted that in the group of healthy women, the concentration of cortisol in milk increases with the decrease in the antioxidant status of milk (FRAP) (Figure 8).

A simultaneous effect of predictors (age, HBD, BMI, lactation period, professional activity, parity, place of residence, occurrence of diseases in breastfeeding women, and type of delivery) on the determined parameters of human milk (DPPH, cortisol, fat, total protein, carbohydrates, dry mass, and caloric value) was observed in the study groups.

## 4. Discussion

There are few studies comparing the composition of milk from women living in different countries, and even fewer analyzing the composition of milk from women living in city centers versus those on the outskirts. Additionally, there is a lack of literature on changes in the antioxidant status of milk in relation to maternal, perinatal, and environmental factors. By reviewing the scientific literature, we decided to investigate the basic composition, cortisol concentration, and antioxidant status of milk from women in Bydgoszcz (Poland), taking into account maternal, perinatal, and environmental factors.

### 4.1. Changes in the Basic Composition of Human Milk in the Context of Many Factors

One of the better-described factors affecting the composition and antioxidant status of human milk is the lactation period. The scientific literature reports that premature milk has a higher concentration of fats, carbohydrates, and calories compared to colostrum. On the other hand, colostrum has a higher concentration of protein, lower fats, and lactose compared to mature milk [7,16,17]. Our studies showed a higher level of total protein in milk from women lactating from 3 to 5 weeks of lactation compared to milk from 5 weeks to 12 months of lactation (*p* = 0.003). In addition, a higher concentration of fats was observed in the milk of women lactating from 5 weeks to 12 months and women lactating > 12 months compared to the milk of women lactating at 3–5 weeks of lactation (*p* ≤ 0.001). Ongprasert et al. (2020) also showed that the level of fats and energy is significantly higher in samples from women who were breastfeeding >12 months [16]. Czosnykowska-Łukacka et al. (2018) observed an increase in fat content, dry matter, and energy value after 12 months of lactation, while after 18 months of lactation, an increase in fat content, dry matter, protein, and energy value was observed, with a simultaneous decrease in carbohydrate content in milk samples [17]. Similar results regarding fat content and energy value were presented by Mandel et al. (2005) [18].

Analyzing the effect of the place of residence in Bydgoszcz, a higher concentration of fats in the milk of women living on the outskirts of the city was noticed compared to the milk of women living in the city center (3.2 vs. 1.9 g/dL; *p* = 0.031). In other studies comparing the milk of Nigerian women and women from Nepal (rural districts), a lower average content of linoleic acid in the milk of Nepalese women was confirmed [19]. Quinn et al. (2016), studying the milk of women living in villages located at high and low altitudes and in a city in Nepal, did not observe any changes in the basal composition of food between the studied groups [20]. Qian et al.’s 2010 study on the content of macro- and micronutrients in the milk of breastfeeding women living in suburban cities and suburban districts of Shanghai showed lower concentrations of proteins, lipids, and higher concentrations of carbohydrates in milk taken from women living in suburban areas [21]. The composition of the milk of Egyptian mothers living in the countryside did not differ significantly in the content of fatty acids, with the exception of linolenic acid, which was not detected in samples of milk obtained from women living in cities [22]. The above-mentioned scientific publications are the only studies showing the variability in the composition of human milk, taking into account the place of residence as a factor determining membership in the study group.

As of today, there are few studies comparing the composition of human milk between groups of women living in different countries. Taking into account the entire study population from Bydgoszcz, it was shown that the fat content in women’s milk is at the level of 2.2 g/dL. Taking into account the literature data, it can be estimated that the level of fat in the milk of Bydgoszcz women is average. Studies show that the basic composition may vary depending on the country of residence of the breastfeeding woman. Comparing the milk of Australian women with the milk of Cambodian mothers, the researchers showed a significantly lower level of total fat (2.90 vs. 3.45 g/dL; *p* = 0.028) and a lower proportion of linoleic acid (9.30% vs. 10.66%, *p* < 0.0001) and α-linolenic acid (0.42% vs. 0.95%, *p* < 0.0001) in the milk obtained from Australian women [23].

Analyzing the data from the previously published studies on the composition of fatty acids in human milk, it can be seen that the DHA content is higher in the milk of women living in countries such as Japan, the Philippines, and Italy, compared to the milk of mothers from the United States, Israel, Tanzania, and the Netherlands. The variability in DHA content may result from the varied diet of breastfeeding women, while its level depends mainly on the amount of seafood consumed [24,25,26,27,28,29,30]. The content of trans fatty acids was lower in the milk of women from Brazil, Germany, and France compared to the group of women from the United States or Canada. These analyses may indicate the consumption of large amounts of processed products by the population of North America [31,32,33]. The differences may be related to the different dietary patterns of these mothers in different cities/countries.

Our research shows that the level of fat in human milk is also affected by fertility (multiparous vs. primiparous), past diseases by the breastfeeding woman, and the type of childbirth. Higher concentrations of fats in primiparous milk, women giving birth through vaginal birth, and suffering from chronic diseases were observed compared to the control groups. Different results were presented by Burianova et al. (2019), who observed a higher protein content (*p* = 0.001) and a lower carbohydrate content (*p* = 0.022) in primiparous milk compared to milk from a multiparous woman. Additionally, protein and carbohydrate levels have been found to be higher at vaginal delivery compared to cesarean section (*p* = 0.036, *p* = 0.003, respectively) [34]. Mangel et al. (2017), Boutsikou et al. (2011), and Signore et al. (2010) did not confirm significant differences in the composition of human milk taking into account the type of delivery [35,36,37].

Maternal factors, such as BMI or age, can affect the basic composition of milk. Our research shows that the milk of women with a BMI of 18–25 kg/m^2^ has a higher fat content compared to the milk of women with a higher BMI (*p* = 0.034).

#### Antioxidant Status of Human Milk in the Context of the Analyzed Factors

The study of the antioxidant status of human milk (DPPH, FRAP) showed that there are no differences between the milk of breastfeeding women from 5 weeks to 12 months after delivery and the milk of breastfeeding women >12 months. On the other hand, higher protection against the DPPH radical was demonstrated in the milk of breastfeeding women from 3 to 5 weeks compared to the other study groups (<0.001). The results were confirmed by Ongprasert et al. (2020), who assessed the total antioxidant capacity of human milk [16]. According to the literature, the antioxidant potential of human milk decreases with the duration of lactation [38,39]. Zarban et al. (2009) showed that the average total antioxidant capacity of colostrum, determined by the FRAP method, is 1061.6 ± 500.6 μM; transitional milk, 915.3 ± 511.4 μM; and mature milk, 724.7 ± 302.4 μM. Researchers also assessed the ability of human milk to reduce the DPPH• radical, showing the following average results: for colostrum, 50.4 ± 19.7%; for transitional milk, 40.8 ± 20.0%; and for mature milk, 38.2 ± 17.3% [40]. Despite the decreasing antioxidant activity of human milk with lactation, human milk is still rich in antioxidants and provides protection against reactive oxygen species, which in turn helps prevent diseases of civilization.

To date, the scientific literature is scarce on the impact of maternal, perinatal, and environmental factors on the antioxidant status of human milk. Our research shows that human milk in women with a BMI of 18–25 kg/m^2^ has greater protection against free radicals compared to the control group (*p* = 0.041).

### 4.2. Cortisol Concentration in Human Milk in the Context of the Analyzed Factors

In the population of the studied women from Bydgoszcz, it was shown that the concentration of cortisol in milk is at the level of 9.2 ng/mL (2017). In human milk, cortisol reaches a concentration of 7.93 ± 4.88 nmol/L, while Hinde et al. (2015) showed a much higher cortisol concentration of 175.5 ± 86.7 nmol/L [41,42]. Cortisol in human milk is an important component due to the regulation of the digestive system and its impact on the child’s neurological development [43,44]. It is believed that the concentration of cortisol in breast milk may be related to the temperament of the infant [44]. The concentration of this hormone depends on the period of lactation, and maternal and perinatal factors [44]. Our research shows the variability in cortisol concentration in milk depending on the place of residence and the lactation period. A higher concentration of this hormone was observed in the milk of women living in the city center compared to the milk of women living on the outskirts (*p* = 0.007) and in the milk of women lactating from the 3rd to the 5th week of lactation compared to the study groups (*p* = 0.001). Kulski et al. confirm our results [45]. There are few studies on the influence of factors on cortisol levels in milk. One recent study is the publication by Lindberg et al. (2020). Scientists have shown that the concentration of cortisol in human milk is higher in the group of primiparous women compared to the group of multiparous women [46,47]. Our research also shows an increase in cortisol levels in primiparous milk, but this is not a significant difference.

Our research has shown many correlations in the study groups (Supplementary Materials Tables S1−S14). One of the most interesting and significant relationships is the negative correlation between fat concentration and cortisol concentration in milk from breastfeeding women from 5 weeks to 12 months postpartum. Linderborg et al. (2022) also investigated similar relationships, demonstrating associations between cortisol in milk and the composition of fatty acids in human milk [47]. Cortisol may be one of the factors influencing lipid origin in human milk. A common finding is the correlation between BMI and fat content in milk. Titi Young et al. (2014) demonstrated a positive association between BMI and fat content in milk. Other maternal factors were not significant for milk composition [48].

Being aware of the simultaneous influence of many factors, we performed multiple linear regressions between the studied parameters (DPPH, cortisol, fat, total protein, carbohydrates, dry matter, and caloric content) and predictors (HBD, BMI, age, lactation period, professional activity, fertility, type of delivery, and residence) in the study groups. We noticed the simultaneous effect of several predictors on the composition and antioxidant status of milk in the study groups.

A limitation of our study is the lack of measurement of the daily volume of milk from the breastfeeding women. However, this study was designed to minimize the effects on breastfeeding in study participants due to the important role of human milk for infants and newborns. The second limitation is the number of participants; therefore, it is worth repeating this study on a larger scale. However, our research is the first to describe the influence of maternal factors and perinatal and environmental effects on the composition and antioxidant status of human milk. Additionally, it is the first work focused on breastfeeding women in Bydgoszcz (Poland).

## 5. Conclusions

The composition and antioxidant status of milk from women from Bydgoszcz (Poland) varies depending on maternal, perinatal, and environmental factors. The basic composition (fat, total protein, and caloric value) is primarily influenced by the period of lactation, parity, place of residence, and professional activity. Higher cortisol concentration in human milk is probably determined by the area of residence (city center and associated higher noise/sound and stress intensity) and the period of lactation (hormonal imbalance, fatigue, and postpartum period).

Milk from professionally active women, compared to the milk of professionally inactive women, shows greater protection against reactive oxygen species, protecting against the occurrence of diseases of civilization. Milk from women breastfeeding a child over 12 months of age also shows protection against reactive oxygen species, despite the fact that the highest level of antioxidant status of human milk occurs in the initial period of lactation.

## Figures and Tables

**Table 1:** General characteristics of breastfeeding women (*n* = 183).

Variable	Median (Q1–Q3)
Age (years)	30 (28–33)
HBD (week)	39 (36–40)
BMI (kg/m^2^)	23.14 (20.98–26.13)
Fat in milk (g/dL)	2.2 (1.5–4.0)
Total protein in milk (g/dL)	1.2 (1.1–1.3)
Carbohydrates in milk (g/dL)	7.9 (7.6–8.2)
Dry Matter (g/dL)	13.1 (12.2–14.2)
Calorie content (kcal/dL)	72 (63–80)
Cortisol (ng/mL)	9.2 (6.03–14.61)
Delivery type	*n* (%)
Vaginal delivery	109 (59%)
Cesarean section	74 (41%)
Place of residence	*n* (%)
City center	108 (60%)
The outskirts of the city	75 (40%)
Maternal parity	*n* (%)
Primiparous	84 (46%)
Multiparous	99 (54%)
Stimulants	*n* (%)
Yes	36 (20%)
No	147 (80%)
Supplementation	*n* (%)
Yes	144 (79%)
No	39 (21%)
Diseases during pregnancy and breastfeeding	*n* (%)
Yes	*n* = 98 (54%)
No	*n* = 85 (46%)
Labor force participation	*n* (%)
Yes	*n* = 109 (60%)
No	*n* = 74 (40%)
Miscarriage	*n* (%)
Yes	33 (18%) including: 1 miscarriage 23 (70%) 2–3 miscarriages 10 (30%)
No	150 (82%)

*n*—size; BMI—body mass index (kg/m^2^); HBD—a week of pregnancy at the time of delivery.

**Table 2:** Characteristics of the study groups.

(**a**)										
	**Group I**			**Group II**			**Group III**			
	**Multiparous**	**Primiparous**	***p***	**Women Living on the Outskirts of the City**	**Women Living in the City Center**	***p***	**Women Giving Birth Naturally**	**Women Giving Birth by Cesarean Section**		***p***
	*n* = 99	*n* = 84		*n* = 75	*n* = 108		*n* = 109	*n* = 74		
Age (years)	32 29–34	29 27–32	<0.001	30 28–32	31 28–34	0.101	30 28–33	30.5 29–34		0.489
BMI (kg/m^2^) (Me; Q25–Q75)	23.6 21.25–26.34	22.55 20.84–25.43	0.150	23.52 20.94–26.13	23.04 20.99–26.13	0.647	22.8 21.2–25.7	23.61 20.96–26.77		0.443
HBD (tyg.) (Me; Q25–Q75)	39 36–40	39 36–40	0.664	39 36–40	39 36–40	0.136	39 38–40	38 32–40		0.004
(**b**)										
	**Group IV**			**Group V**			**Group VI**			
	**Professionally Active Women**	**Professionally Inactive Women**	***p***	**Women who Fell Ill During Pregnancy and Breastfeeding**	**Healthy Women**	***p***	**Breast-feeding from 3 to 5 Weeks**	**Breast-feeding from 5 Weeks up to 12 Months of Lactation**	**Breast-feeding from 12 to 24 Months**	***p_a/b_*** ***p_a/c_*** ***p_b/c_***
	*n* = 111	*n* = 72		*n* = 98	*n* = 85		*n* = 76	*n* = 59	*n* = 48	
Age (years)	31 29–33	29.5 27–32	0.066	30 26–34	30 26–32	0.474	31 28–34	30 28–32	31 29–33	0.612 > 0.999 0.212
BMI (kg/m^2^) (Me; Q25–Q75)	23.53 20.91–26.34	23.04 21.24–25.00	0.634	22.86 20.94–25.82	23.24 21.45–26.35	0.823	24.35 22.18–27.49	22.57 20.89–24.80	21.6 19.61–24.50	0.009 < 0.001 0.853
HBD (tyg.) (Me; Q25–Q75)	39 35–40	39 36–40	0.689	39 36–40	39 35–40	0.863	35 31–39	40 39–41	40 39–40	<0.001 < 0.001 > 0.999

*n*—size; BMI—body mass index (kg/m^2^); HBD—a week of pregnancy at the time of delivery; Me–median; Q25–Q75—lower and upper quartiles; and *p*—level of statistical significance (Mann–Whitney U test).

**Table 3:** Analysis of cortisol concentration, basic composition, and antioxidant status of human milk in the study groups.

(**a**)										
	**Group I**					**Group II**				
	**Multiparous**	**Primiparous**			***p***	**Women Living on the Outskirts of the City**		**Women Living in the City Center**		***p***
	*n* = 99	*n* = 84				*n* = 75		*n* = 108		
Fats (Me; Q25–Q75)	1.9 1.5–3.9	2.65 1.6–4.55			0.040	3.2 1.6–4.2		1.9 1.5–3.95		0.031
Total protein (Me; Q25–Q75)	1.2 1.1–1.3	1.2 1.1–1.4			0.262	1.2 1–1.3		1.2 1.1–1.3		0.450
Carbohydrates (Me; Q25–Q75)	8 7.7–8.2	7.8 7.5–8.1			0.179	7.9 7.5–8.2		7.9 7.6–8.1		0.851
Dry matter (Me; Q25–Q75)	13.2 12.2–14.1	13 12.25–14.3			0.958	13.1 12.4–14.1		13 12.05–14.25		0.688
Caloric value (Me; Q25–Q75)	72 64–80	71.5 63–80.5			0.998	72 65–82		73 62.5–80		0.585
Cortisol [ng/mL] (Me; Q25–Q75)	8.57 5.7–14.35	10.24 6.22–15.41			0.641	8.13 6.84–12.74		10.67 6.87–16.27		0.007
FRAP (Me; Q25–Q75)	758.52 454.2–1108	764.75 461–978.54			0.779	729.75 449.65–1053.75		776.68 461.76–1026.5		0.570
DPPH (Me; Q25–Q75)	57.64 50.99–67.23	56.5 47.26–72.17			0.731	56.53 50.54–68.5		57.37 48.38–71.53		0.910
	**Group III**					**Group IV**				
	**Women Giving Birth Naturally**			**Women Giving Birth by Cesarean Section**	***p***	**Professionally Active Women**		**Professionally Inactive Women**		***p***
	*n* = 109			*n* = 74		*n* = 111		*n* = 72		
Fats (Me; Q25–Q75)	3.2 1.6–4.4			1.85 1.5–3.2	0.009	2 1.5–3.9		3.05 1.55–4.15		0.176
Total protein (Me; Q25–Q75)	1.2 1.1–1.3			1.2 1.1–1.4	0.491	1.2 1.1–1.4		1.1 1–1.3		0.002
Carbohydrates (Me; Q25–Q75)	8 7.6–8.2			7.9 7.5–8.1	0.366	7.9 7.6–8.2		7.9 7.5–8.1		0.196
Dry matter (Me; Q25–Q75)	13.4 12.4–14.3			12.8 12–13.4	0.041	13.5 12.4–14.5		12.8 11.95–13.95		0.046
Caloric value (Me; Q25–Q75)	73 62–79			69.5 62–79	0.127	74 65–82		69 61–78		0.087
Cortisol [ng/mL] (Me; Q25–Q75)	8.83 6.02–18.29			11 6.25–18.29	0.177	9.55 6.25–13.79		8.7 5.06–15.45		0.586
FRAP (Me; Q25–Q75)	758.52 461.77–1058.09			767.65 454.2–984.8	0.944	708.55 445.12–908.40		806.21 522.18–1120.36		0.146
DPPH (Me; Q25–Q75)	56.4 47.83–67.87			58.46 52.26–69.55	0.259	53.01 42.61–66.10		58.42 53.27–71.32		0.002
(**b**)										
**Group V**				**Group VI**				**Group VII**		
	**Women Who Fell Ill During Pregnancy and Breastfeeding**	**Healthy Women**	***p***	**Breastfeeding from 3 to 5 Weeks**	**Breastfeeding from 5 Weeks up to 12 Months of Lactation**	**Breastfeeding from 12 to 24 Months**	***p^a/b^*** ***p_a/c_*** ***p^b/c^***	**Women with a BMI of 18–25 kg/m^2^**	**Women with a BMI of 25–35 kg/m^2^**	***p***
	*n* = 98	*n* = 85		*n* = 76	*n* = 59	*n* = 48		*n* = 110	*n* = 73	
Fats (Me; Q25–Q75)	3.05 1.6–4.4	1.8 1.5–3.4	0.005	1.5 1.4–1.7	3.4 2.6–4.4	4.4 3.2–6.1	<0.001 < 0.001 0.103	2.8 1.6–4.3	1.85 1.5–3.3	0.034
Total protein (Me; Q25–Q75)	1.2 1.1–1.3	1.2 1.1–1.3	0.395	1.2 1.1–1.4	1.1 1.0–1.2	1.2 1.1–1.5	0.003 > 0.999 0.087	1.2 1.1–1.3	1.2 1.1–1.3	0.465
Carbohydrates (Me; Q25–Q75)	7.9 7.6–8.1	7.9 7.6–8.2	0.964	7.9 7.5–8.3	8 7.8–8.1	7.9 7.4–8.1	>0.999 0.107 0.127	7.9 7.6–8.1	7.9 7.6–8.2	0.237
Dry matter (Me; Q25–Q75)	13.05 12.2–14.5	13.1 12.2–14.1	0.461	13.1 12.5–14	12.5 11.7–13.9	13.45 12.1–15.2	0.158 > 0.999 0.042	13 12.1–13.9	13.3 12.4–14.8	0.245
Caloric value (Me; Q25–Q75)	71 63–86	73 64–80	0.564	74 98–79.5	67 60–77	77 64–90	0.016 > 0.999 0.004	72 63–78	73.5 64–86	0.223
Cortisol [ng/mL] (Me; Q25–Q75)	8.86 5.81–13.58	9.65 6.26–15.31	0.434	13.21 9.18–19.47	6.1 3.9–10.9	8.48 4.9–11	<0.001 < 0.001 0.956	9.08 5.69–14.61	9.43 6.36–13.32	0.671
FRAP (Me; Q25–Q75)	702.74 472.36–984.10	635.88 445.11–1114.39	0.433	887.7 608.6–115.82	582.37 374.5–935.4	775.25 461.97–947.67	0.001 0.489 0.267	699.47 454.2–984.1	778.95 461.17–1058	0.535
DPPH (Me; Q25–Q75)	58.09 48.99–72.05	56.47 49.11–67.32	0.404	62.67 55.21–74.01	50.25 43.20–60.00	54.40 47.32–66.00	<0.001 0.002 0.877	58.42 53.27–71.32	55.99 47.23–68.05	0.041

*n*—size; Me—median; Q25–Q75—lower and upper quartiles; *p*—level of statistical significance (Mann–Whitney U test); FRAP—iron ion reduction ability; and DPPH—radical 2.2-diphenyl-1-picrylhydrazyl.

## Data Availability

The original contributions presented in the study are included in the article/Supplementary Materials, further inquiries can be directed to the corresponding author.

## Supplementary Materials

The following supporting information can be downloaded at: https://www.mdpi.com/article/10.3390/nu16193396/s1, Table S1. Spearman rank order correlations for the root group, Table S2. Spearman rank order correlations for the multiparous group, Table S3. Correlations of the Spearman rank order for a group of women giving birth naturally, Table S4. Correlations of the Spearman rank order for a group of women giving birth by caesarean section, Table S5. Correlations of Spearman’s rank order for a group of women living in the city center, Table S6. Correlations of Spearman’s rank order for a group of women living on the outskirts of the city, Table S7. Correlations of the Spearman rank order for the group of women breastfeeding with transitional milk, Table S8. Correlations of the Spearman rank order for the group of breastfeeding women under 12 months, Table S9. Correlations of the Spearman rank order for the group of lactating women 12–24 months, Table S10. Correlations of the Spearman rank order for the group of women who did not suffer during pregnancy and breastfeeding, Table S11. Correlations of the Spearman rank order for a group of women suffering from the disease during pregnancy and breastfeeding, Table S12. Correlations of the Spearman rank order for the group of professionally active women, Table S13. Correlations of the Spearman rank order for the group of professionally inactive women, Table S14. Results of multiple linear regression between the studied parameters (DPPH, FRAP, cortisol, fat, total protein, carbohydrates, dry mass, caloric value) and predictors (HBD, BMI, age, lactation phases, professional activity, parity, type of delivery, place of residence) in the study groups.
