# Supplementary material for: Composition and Antioxidant Status of Human Milk of Women Living in Bydgoszcz (Poland)
Source: Nutrients. 2024 Oct 6;16(19):3396. doi: 10.3390/nu16193396 (PMC11479156; doi:10.3390/nu16193396)
Supplement: Supplementary file 1 [file nutrients-16-03396-s001.zip › nutrients-3207865-supplementary.pdf]

SUPPLEMENTARY MATERIALS

Table S1 Spearman rank order correlations for the root group

Variable	HBD	Age	BMI	Cortisol	DPPH	FRAP	fat	Total protein	carbohydrates	dry matter	Caloric value
HBD	1.000	0.111	-0.001	-0.204	-0.290	0.012	0.338	-0.090	0.124	-0.048	-0.148
Age	0.111	1.000	0.079	-0.169	0.007	-0.079	0.094	-0.146	0.072	0.043	-0.022
BMI	-0.001	0.079	1.000	0.174	0.225	-0.060	-0.234	0.084	0.007	0.031	0.071
Cortisol	-0.204	-0.169	0.174	1.000	0.046	-0.071	-0.314	0.113	0.092	0.117	0.193
DPPH	-0.290	0.007	0.225	0.046	1.000	-0.130	-0.023	0.155	0.010	0.290	0.3185
FRAP	0.012	-0.079	-0.060	-0.071	-0.130	1.000	0.106	-0.040	0.063	-0.064	-0.070
Fat	0.338	0.094	-0.234	-0.314	-0.023	0.106	1.000	0.156	-0.031	0.460	0.389
Total protein	-0.090	-0.146	0.084	0.113	0.155	-0.040	0.156	1.000	-0.132	0.437	0.389
Carbohydrates	0.124	0.072	0.007	0.092	0.010	0.063	-0.031	-0.132	1.000	0.212	0.077
dry matter	-0.048	0.043	0.031	0.117	0.290	-0.064	0.460	0.437	0.212	1.000	0.906

BMI – body mass index; FRAP – iron ion reduction capacity [μM]; DPPH – 2.2-diphenyl-1-picrylhydrazyl radical [% inhibition]; cortisol – concentration in ng/ml; fat. total protein. carbohydrates. dry matter – content in human milk in g/100ml; energy value – content in kcal/100ml

Table S2 Spearman rank order correlations for the multiparous group

Variable	HBD	Age	BMI	cortisol	DPPH	FRAP	Fat	Total protein	carbohydrates	dry matter	Caloric value
HBD	1.000	-0.136	-0.255	-0.192	-0.421	0.162	0.278	-0.092	-0.145	-0.096	-0.068
Age	-0.136	1.000	-0.077	0.201	-0.079	-0.080	0.062	0.019	0.053	0.021	-0.023
BMI	-0.255	-0.075	1.000	-0.088	0.171	0.079	-0.175	-0.041	-0.011	0.040	0.053
Cortisol	-0.192	0.201	-0.088	1.000	-0.001	-0.318	-0.269	0.106	0.083	0.021	0.013
DPPH	-0.421	-0.079	0.171	-0.001	1.000	-0.158	-0.320	0.206	0.049	0.108	0.138
FRAP	0.162	-0.002	0.079	-0.318	-0.158	1.000	0.108	0.087	0.027	-0.035	-0.092
Fat	0.278	0.062	-0.175	-0.269	-0.320	0.108	1.000	0.089	0.013	0.456	0.398
Total protein	-0.092	0.019	-0.041	0.106	0.206	0.087	0.089	1.000	0.250	0.332	0.226
Carbohydrates	-0.145	0.053	-0.011	0.083	0.049	0.027	0.013	0.250	1.000	0.347	0.092

dry matter	-0.096	0.021	0.040	0.021	0.108	-0.035	0.456	0.332	0.347	1.000	0.882

BMI – body mass index; FRAP – iron ion reduction capacity [μM]; DPPH – 2.2-diphenyl-1-picrylhydrazyl radical [% inhibition]; cortisol – concentration in ng/ml; fat. total protein. carbohydrates. dry matter – content in human milk in g/100ml; energy value – content in kcal/100ml

Table S3 Correlations of the Spearman rank order for a group of women giving birth naturally

Variable	HBD	Age	BMI	cortisol	DPPH	FRAP	fat	Total protein	carbohydrates	dry matter	Caloric value
HBD	1.000	-0.100	-0.155	-0.099	-0.288	0.032	0.206	-0.142	-0.098	-0.186	-0.162
Age	-0.100	1.000	-0.037	0.101	-0.049	-0.220	0.088	-0.129	0.006	0.105	0.048
BMI	-0.155	-0.037	1.000	-0.009	0.201	0.073	-0.163	0.070	-0.077	0.035	0.072
Cortisol	-0.099	0.101	-0.009	1.000	-0.019	-0.165	-0.201	0.226	-0.083	0.115	0.202
DPPH	-0.288	-0.049	0.201	-0.019	1.000	-0.124	-0.173	0.248	0.121	0.244	0.228
FRAP	0.032	-0.220	0.073	-0.165	-0.124	1.000	0.078	0.139	0.042	-0.077	-0.140
Fat	0.206	0.088	-0.163	-0.201	-0.173	0.078	1.000	0.067	-0.110	0.477	0.404
Total protein	-0.142	-0.129	0.070	0.226	0.248	0.139	0.067	1.000	0.073	0.347	0.285
Carbohydrates	-0.098	0.006	-0.077	-0.083	0.121	0.042	-0.110	0.073	1.000	0.257	0.066
dry matter	-0.186	0.105	0.035	0.115	0.244	-0.077	0.477	0.347	0.257	1.000	0.865

BMI – body mass index; FRAP – iron ion reduction capacity [μM]; DPPH – 2.2-diphenyl-1-picrylhydrazyl radical [% inhibition]; cortisol – concentration in ng/ml; fat. total protein. carbohydrates. dry matter – content in human milk in g/100ml; energy value – content in kcal/100ml

Table S4 Correlations of the Spearman rank order for a group of women giving birth by caesarean section

Variable	HBD	Age	BMI	cortisol	DPPH	FRAP	fat	Total protein	carbohydrates	dry matter	Caloric value
HBD	1.000	0.095	-0.120	-0.229	-0.417	0.200	0.369	0.016	0.082	-0.016	-0.119
Age	0.095	1.000	0.078	-0.058	-0.036	0.158	-0.019	-0.028	0.193	-0.021	-0.090
BMI	-0.120	0.078	1.000	0.052	0.187	-0.068	-0.234	-0.087	0.086	0.067	0.091
Cortisol	-0.229	-0.058	0.052	1.000	0.025	-0.224	-0.357	-0.053	0.285	0.027	-0.014
DPPH	-0.417	-0.036	0.187	0.025	1.000	-0.179	-0.144	0.076	-0.113	0.188	0.283
FRAP	0.200	0.158	-0.068	-0.224	-0.179	1.000	0.141	-0.110	0.092	-0.025	-0.026
Fat	0.369	-0.019	-0.234	-0.357	-0.144	0.141	1.000	0.276	0.041	0.387	0.334
Total protein	0.016	-0.028	-0.087	-0.053	0.076	-0.110	0.276	1.000	0.062	0.479	0.397
Carbohydrates	0.082	0.193	0.086	0.285	-0.113	0.092	0.041	0.062	1.000	0.322	0.088
dry matter	-0.016	-0.021	0.067	0.027	0.188	-0.025	0.387	0.479	0.322	1.000	0.937

BMI – body mass index; FRAP – iron ion reduction capacity [μM]; DPPH – 2.2-diphenyl-1-picrylhydrazyl radical [% inhibition]; cortisol – concentration in ng/ml; fat. total protein. carbohydrates. dry matter – content in human milk in g/100ml; energy value – content in kcal/100ml

Table S5 Correlations of Spearman's rank order for a group of women living in the city center

Variable	HBD	Age	BMI	cortisol	DPPH	FRAP	Fat	Total protein	carbohydrates	dry matter	Caloric value
HBD	1.000	0.014	-0.105	-0.289	-0.380	0.083	0.271	-0.152	0.025	-0.039	-0.076
Age	0.014	1.000	-0.004	-0.007	-0.127	-0.005	0.042	-0.120	0.219	0.007	-0.060
BMI	-0.105	-0.004	1.000	-0.022	0.166	0.063	-0.215	-0.044	-0.046	0.019	0.075
Cortisol	-0.289	-0.007	-0.022	1.000	0.042	-0.190	-0.251	0.070	0.134	0.087	0.113
DPPH	-0.380	-0.127	0.166	0.042	1.000	0.030	-0.135	0.337	0.101	0.260	0.231
FRAP	0.083	-0.005	0.063	-0.190	0.030	1.000	0.044	0.037	0.115	0.027	0.019
Fat	0.271	0.042	-0.215	-0.251	-0.135	0.044	1.000	0.233	-0.074	0.428	0.429
Total protein	-0.152	-0.120	-0.044	0.070	0.337	0.037	0.233	1.000	-0.024	0.346	0.338
Carbohydrates	0.025	0.219	-0.046	0.134	0.101	0.115	-0.074	-0.024	1.000	0.322	0.132
dry matter	-0.039	0.007	0.019	0.087	0.260	0.027	0.428	0.346	0.322	1.000	0.936

BMI – body mass index; FRAP – iron ion reduction capacity [μM]; DPPH – 2.2-diphenyl-1-picrylhydrazyl radical [% inhibition]; cortisol – concentration in ng/ml; fat. total protein. carbohydrates. dry matter – content in human milk in g/100ml; energy value – content in kcal/100ml

Table S6 Correlations of Spearman's rank order for a group of women living on the outskirts of the city

Variable	HBD	Age	BMI	cortisol	DPPH	FRAP	fat	Total protein	carbohydrates	dry matter	Caloric value
HBD	1.000	-0.137	-0.189	-0.129	-0.325	0.145	0.400	-0.077	-0.088	-0.139	-0.155
Age	-0.137	1.000	0.075	0.104	0.132	-0.146	0.094	-0.046	-0.152	0.133	0.119
BMI	-0.189	0.075	1.000	0.186	0.242	-0.088	-0.213	0.090	0.084	0.077	0.066
Cortisol	-0.129	0.104	0.186	1.000	0.009	-0.239	-0.304	0.119	-0.026	0.026	0.067
DPPH	-0.325	0.132	0.242	0.009	1.000	-0.398	-0.245	-0.001	-0.061	0.129	0.240
FRAP	0.145	-0.146	-0.088	-0.239	-0.398	1.000	0.174	0.042	-0.023	-0.174	-0.269
Fat	0.400	0.094	-0.213	-0.304	-0.245	0.174	1.000	0.077	0.004	0.519	0.379
Total protein	-0.077	-0.046	0.090	0.119	-0.001	0.042	0.077	1.000	0.155	0.473	0.315
Carbohydrates	-0.088	-0.152	0.084	-0.026	-0.061	-0.023	0.004	0.155	1.000	0.248	0.014
dry matter	-0.139	0.133	0.077	0.026	0.129	-0.174	0.519	0.473	0.248	1.000	0.835

BMI – body mass index; FRAP – iron ion reduction capacity [μM]; DPPH – 2.2-diphenyl-1-picrylhydrazyl radical [% inhibition]; cortisol – concentration in ng/ml; fat. total protein. carbohydrates. dry matter – content in human milk in g/100ml; energy value – content in kcal/100ml

Table S7 Correlations of the Spearman rank order for the group of women breastfeeding with transitional milk

Variable	HBD	Age	BMI	cortisol	DPPH	FRAP	fat	Total protein	carbohydrates	dry matter	Caloric value
HBD	1.000	0.051	-0.022	-0.156	-0.276	0.147	0.020	0.010	0.046	0.109	0.116
Age	0.051	1.000	-0.060	0.041	-0.141	0.044	0.036	-0.018	0.308	0.013	-0.141
BMI	-0.022	-0.060	1.000	-0.285	-0.021	0.107	0.029	0.056	-0.114	0.045	0.048
Cortisol	-0.156	0.041	-0.285	1.000	-0.216	-0.108	-0.030	-0.003	0.114	-0.010	-0.097
DPPH	-0.276	-0.141	-0.021	-0.216	1.000	-0.111	0.022	0.084	-0.064	-0.013	0.067
FRAP	0.147	0.044	0.107	-0.108	-0.111	1.000	0.095	0.124	0.071	0.134	0.114
Fat	0.020	0.036	0.029	-0.030	0.022	0.095	1.000	0.951	0.082	0.298	0.157
Total protein	0.010	-0.018	0.056	-0.003	0.084	0.124	0.951	1.000	0.066	0.311	0.176
Carbohydrates	0.046	0.308	-0.114	0.114	-0.064	0.071	0.082	0.066	1.000	0.420	-0.038
dry matter	0.109	0.013	0.045	-0.010	-0.013	0.134	0.298	0.311	0.420	1.000	0.804

BMI – body mass index; FRAP – iron ion reduction capacity [μM]; DPPH – 2.2-diphenyl-1-picrylhydrazyl radical [% inhibition]; cortisol – concentration in ng/ml; fat. total protein. carbohydrates. dry matter – content in human milk in g/100ml; energy value – content in kcal/100ml

Table S8 Correlations of the Spearman rank order for the group of breastfeeding women under 12 months

Variable	HBD	Age	BMI	cortisol	DPPH	FRAP	fat	Total protein	carbohydrates	dry matter	Caloric value
HBD	1.000	-0.034	0.189	0.124	-0.275	-0.137	-0.212	0.006	-0.223	-0.279	-0.260
Age	-0.034	1.000	0.092	-0.168	0.000	-0.097	-0.047	-0.411	-0.335	-0.135	-0.148
BMI	0.189	0.092	1.000	0.088	0.304	0.000	0.090	-0.055	0.013	0.076	0.114
Cortisol	0.124	-0.168	0.088	1.000	-0.186	-0.099	0.074	0.173	-0.004	0.048	0.135
DPPH	-0.275	0.000	0.304	-0.186	1.000	-0.092	0.260	-0.008	0.106	0.301	0.231
FRAP	-0.137	-0.097	0.000	-0.099	-0.092	1.000	-0.094	0.252	0.071	-0.061	-0.048
Fat	-0.212	-0.047	0.090	0.074	0.260	-0.094	1.000	0.076	0.213	0.965	0.877
Total protein	0.006	-0.411	-0.055	0.173	-0.008	0.252	0.076	1.000	0.225	0.203	0.127
Carbohydrates	-0.223	-0.335	0.013	-0.004	0.106	0.071	0.213	0.225	1.000	0.374	0.413
dry matter	-0.279	-0.135	0.076	0.048	0.301	-0.061	0.965	0.203	0.374	1.000	0.903

BMI – body mass index; FRAP – iron ion reduction capacity [μM]; DPPH – 2.2-diphenyl-1-picrylhydrazyl radical [% inhibition]; cortisol – concentration in ng/ml; fat. total protein. carbohydrates. dry matter – content in human milk in g/100ml; energy value – content in kcal/100ml

Table S9 Correlations of the Spearman rank order for the group of lactating women 12-24 months

Variable	HBD	Age	BMI	cortisol	DPPH	FRAP	fat	Total protein	carbohydrates	dry matter	Caloric value
HBD	1.000	-0.071	-0.099	0.374	-0.041	-0.238	-0.014	-0.069	0.111	0.028	-0.018
Age	-0.071	1.000	0.121	0.264	-0.028	-0.175	0.243	0.059	0.078	0.260	0.241
BMI	-0.099	0.121	1.000	-0.142	0.036	0.137	0.055	-0.133	-0.065	0.006	0.045
Cortisol	0.374	0.264	-0.142	1.000	-0.128	-0.239	0.045	-0.259	0.146	0.034	0.031
DPPH	-0.041	-0.028	0.036	-0.128	1.000	0.080	0.177	0.265	0.007	0.245	0.244
FRAP	-0.238	-0.175	0.137	-0.239	0.080	1.000	-0.297	-0.070	-0.037	-0.274	-0.289
Fat	-0.014	0.243	0.055	0.045	0.177	-0.297	1.000	0.402	-0.079	0.937	0.976
Total protein	-0.069	0.059	-0.133	-0.259	0.265	-0.070	0.402	1.000	-0.027	0.543	0.510
Carbohydrates	0.111	0.078	-0.065	0.146	0.007	-0.037	-0.079	-0.027	1.000	0.172	0.012
dry matter	0.028	0.260	0.006	0.034	0.245	-0.274	0.937	0.543	0.172	1.000	0.967

BMI – body mass index; FRAP – iron ion reduction capacity [μM]; DPPH – 2.2-diphenyl-1-picrylhydrazyl radical [% inhibition]; cortisol – concentration in ng/ml; fat. total protein. carbohydrates. dry matter – content in human milk in g/100ml; energy value – content in kcal/100ml

Table S10 Correlations of the Spearman rank order for the group of women who did not suffer during pregnancy and breastfeeding

Variable	HBD	Age	BMI	cortisol	DPPH	FRAP	fat	Total protein	carbohydrates	dry matter	Caloric value
HBD	1.000	0.101	0.021	-0.282	-0.300	0.073	0.244	-0.101	-0.065	-0.154	-0.168
Age	0.101	1.000	-0.161	-0.096	-0.187	-0.051	0.093	-0.163	0.127	-0.033	-0.171
BMI	0.021	-0.161	1.000	0.043	-0.030	0.080	0.015	0.060	-0.055	-0.008	-0.018
Cortisol	-0.282	-0.096	0.043	1.000	0.060	-0.421	-0.349	0.007	0.074	0.117	0.140
DPPH	-0.300	-0.187	-0.030	0.060	1.000	-0.049	-0.234	0.193	0.028	0.064	0.107
FRAP	0.073	-0.051	0.080	-0.421	-0.049	1.000	0.123	0.051	0.113	0.008	-0.065
Fat	0.244	0.093	0.015	-0.349	-0.234	0.123	1.000	0.163	-0.025	0.331	0.254
Total protein	-0.101	-0.163	0.060	0.007	0.193	0.051	0.163	1.000	0.003	0.414	0.349
Carbohydrates	-0.065	0.127	-0.055	0.074	0.028	0.113	-0.025	0.003	1.000	0.338	0.062
dry matter	-0.154	-0.033	-0.008	0.117	0.064	0.008	0.331	0.414	0.338	1.000	0.852

BMI – body mass index; FRAP – iron ion reduction capacity [μM]; DPPH – 2.2-diphenyl-1-picrylhydrazyl radical [% inhibition]; cortisol – concentration in ng/ml; fat. total protein. carbohydrates. dry matter – content in human milk in g/100ml; energy value – content in kcal/100ml

Table S11 Correlations of the Spearman rank order for a group of women suffering from the disease during pregnancy and breastfeeding

Variable	HBD	Age	BMI	cortisol	DPPH	FRAP	fat	Total protein	carbohydrates	dry matter	Caloric value
HBD	1.000	-0.133	-0.263	-0.077	-0.397	0.095	0.368	-0.081	0.042	0.015	-0.054
Age	-0.133	1.000	0.135	0.141	0.070	-0.096	-0.064	-0.021	0.054	0.091	0.090
BMI	-0.263	0.135	1.000	0.013	0.380	-0.026	-0.351	-0.029	0.031	0.068	0.123
Cortisol	-0.077	0.141	0.013	1.000	-0.003	0.011	-0.249	0.191	0.071	0.008	0.023
DPPH	-0.397	0.070	0.380	-0.003	1.000	-0.206	-0.147	0.188	0.016	0.295	0.331
FRAP	0.095	-0.096	-0.026	0.011	-0.206	1.000	0.066	0.011	0.011	-0.087	-0.089
Fat	0.368	-0.064	-0.351	-0.249	-0.147	0.066	1.000	0.086	-0.050	0.559	0.507
Total protein	-0.081	-0.021	-0.029	0.191	0.188	0.011	0.086	1.000	0.104	0.361	0.298
Carbohydrates	0.042	0.054	0.031	0.071	0.016	0.011	-0.050	0.104	1.000	0.248	0.078
dry matter	0.015	0.091	0.068	0.008	0.295	-0.087	0.559	0.361	0.248	1.000	0.921

BMI – body mass index; FRAP – iron ion reduction capacity [μM]; DPPH – 2.2-diphenyl-1-picrylhydrazyl radical [% inhibition]; cortisol – concentration in ng/ml; fat. total protein. carbohydrates. dry matter – content in human milk in g/100ml; energy value – content in kcal/100ml

Table S12 Correlations of the Spearman rank order for the group of professionally active women

Variable	HBD	Age	BMI	cortisol	DPPH	FRAP	fat	Total protein	carbohydrates	dry matter	Caloric value
HBD	1.000	-0.065	-0.129	-0.093	-0.340	0.076	0.277	-0.080	-0.018	0.040	0.022
Age	-0.065	1.000	-0.015	0.226	0.021	-0.036	0.064	0.067	0.145	0.159	0.119
BMI	-0.129	-0.015	1.000	0.083	0.086	0.097	-0.297	-0.023	0.031	-0.023	-0.017
Cortisol	-0.093	0.226	0.083	1.000	-0.007	-0.166	-0.193	0.062	0.137	0.106	0.100
DPPH	-0.340	0.021	0.086	-0.007	1.000	-0.065	-0.236	0.176	0.013	0.076	0.080
FRAP	0.076	-0.036	0.097	-0.166	-0.065	1.000	0.004	-0.021	0.048	-0.189	-0.192
Fat	0.277	0.064	-0.297	-0.193	-0.236	0.004	1.000	0.209	-0.100	0.441	0.462
Total protein	-0.080	0.067	-0.023	0.062	0.176	-0.021	0.209	1.000	-0.036	0.416	0.395
Carbohydrates	-0.018	0.145	0.031	0.137	0.013	0.048	-0.100	-0.036	1.000	0.242	0.007
dry matter	0.040	0.159	-0.023	0.106	0.076	-0.189	0.441	0.416	0.242	1.000	0.951

BMI – body mass index; FRAP – iron ion reduction capacity [μM]; DPPH – 2.2-diphenyl-1-picrylhydrazyl radical [% inhibition]; cortisol – concentration in ng/ml; fat. total protein. carbohydrates. dry matter – content in human milk in g/100ml; energy value – content in kcal/100ml

Table S13 Correlations of the Spearman rank order for the group of professionally inactive women

Variable	HBD	Age	BMI	Cortisol	DPPH	FRAP	fat	Total protein	carbohydrates	dry matter	Caloric value
HBD	1.00	0.05	-0.11	-0.32	-0.37	0.12	0.29	-0.09	-0.02	-0.24	-0.30
Age	0.05	1.00	0.07	-0.27	-0.16	-0.07	0.01	-0.42	-0.05	-0.19	-0.29
BMI	-0.11	0.07	1.00	0.00	0.37	-0.06	-0.02	0.03	-0.04	0.18	0.23
Cortisol	-0.32	-0.27	0.00	1.00	0.07	-0.26	-0.41	0.13	-0.01	-0.06	0.03
DPPH	-0.37	-0.16	0.37	0.07	1.00	-0.18	-0.04	0.08	0.03	0.31	0.36
FRAP	0.12	-0.07	-0.06	-0.26	-0.18	1.00	0.21	0.17	0.08	0.19	0.08
Fat	0.29	0.01	-0.02	-0.41	-0.04	0.21	1.00	0.14	0.06	0.55	0.37
Total protein	-0.09	-0.42	0.03	0.13	0.08	0.17	0.14	1.00	0.18	0.35	0.16
Carbohydrates	-0.02	-0.05	-0.04	-0.01	0.03	0.08	0.06	0.18	1.00	0.35	0.13
dry matter	-0.24	-0.19	0.18	-0.06	0.31	0.19	0.55	0.35	0.35	1.00	0.78

BMI – body mass index; FRAP – iron ion reduction capacity [μM]; DPPH – 2.2-diphenyl-1-picrylhydrazyl radical [% inhibition]; cortisol – concentration in ng/ml; fat. total protein. carbohydrates. dry matter – content in human milk in g/100ml; energy value – content in kcal/100ml

Table S14. Results of multiple linear regression between the studied parameters (DPPH, FRAP, cortisol, fat, total protein, carbohydrates, dry mass, caloric value) and predictors (HBD, BMI, age, lactation phases, professional activity, parity, type of delivery, place of residence) in the study groups.

**GROUP 1A**		**DPPH**	**Cortisol**	**Fat**	**Total Protein**	**Carbohydrates**	**GROUP 1B**	**DPPH**	**Fat**	**Total protein**	**Dry mass**	**Energy value**
**HBD**	B		-0.313					-0.277				
	HERSELF		0.119					0.079				
	P		0.010					0.001				
**BMI**	B								0.115	0.308	0.309	0.328
	HERSELF								0.097	0.121	0.112	0.118
	P								0.048	0.013	0.012	0.007
**Period**	B	-0.256		0.637				-0.191	0.612			0.287
**Lactation**	HERSELF	0.107		0.082				0.080	0.118			0.143
	P	0.013		<0.001				0.018	0.001			0.048
**Activity**	B				0.375	0.277		0.197				
**Professional**	HERSELF				0.098	0.100		0.067				
	P				0.001	0.007		0.003				
**GROUP 2C**		**DPPH**	**FRAP**	**Cortisol**	**Fat**	**Total Protein**	**GROUP 2D**	**DPPH**	**Cortisol**	**Fat**		
**HBD**	B	-0.349						-0.261	-0.335			

	HERSELF	0.118							0.102	0.100			
	P	0.004							0.011	0.002			
**BMI**	B		0.299										
	HERSELF		0.127										
	P		0.022										
**Period Lactation**	B	-0.240	0.279	-0.288	0.478						0.698		
	HERSELF	0.118	0.139	0.137	0.115						0.087		
	P	0.045	0.049	0.038	0.001						<0.001		
**Activity Professional**	B	0.223											
	HERSELF	0.106											
	P	0.036											
**GROUP 3E**		**DPPH**	**FRAP**	**Cortisol**	**Fat**	**Carbohydrates**	**Dry mass**	**GROUP 3F**	**DPPH**	**FRAP**	**Fat**	**Carbohydrates**	**Cortisol**
**HBD**	B	-0.216		-0.237					-0.355			0.278	
	HERSELF	0.098		0.109					0.123			0.131	
	P	0.030		0.032					0.005			0.039	
**Age**	B		-0.232							0.334		0.291	
	HERSELF		0.099							0.124		0.120	
	P		0.021							0.009		0.018	
**Period Lactation**	B	-0.263			0.624						0.580	-0.309	-0.309
	HERSELF	0.100			0.086						0.116	0.139	0.142
	P	0.010			<0.001						0.001	0.029	0.007
**Activity Professional**	B	0.205				0.254	0.215						
	HERSELF	0.087				0.099	0.097						
	P	0.022				0.011	0.029						
**Fertility**	B								0.273				
	HERSELF								0.110				
	P								0.015				
**GROUP 4G**		**DPPH**	**Fat**	**Carbohydrates**	**Dry mass**	**Energy value**		**GROUP 4H**	**DPPH**	**Cortisol**	**Total protein**		**Fat**
**HBD**	B	-0.298											
	HERSELF	0.102											
	P	0.004											
**Age**	B			0.211	0.212								
	HERSELF			0.100	0.101								
	P			0.037	0.039								
**Type of birth**	B		0.149		0.223	0.219							
	HERSELF		0.066		0.097	0.096							
	P		0.027		0.023	0.025							
**Disease**	B	0.191											
	HERSELF	0.094											

	P	0.044									
**Lactation period**	B	-0.245	0.661	-0.244							0.463
	HERSELF	0.108	0.078	0.113							0.123
	P	0.025	0.001	0.032							0.001
**BMI**	B									0.294	0.271
	HERSELF									0.116	0.128
	P									0.014	0.039
**Fertility**	B									-0.325	-0.292
	HERSELF									0.141	0.128
	P									0.024	0.026
**GROUP 5I**		**DPPH**	**Cortisol**	**Fat**			**GROUP 5J**	**DPPH**	**Cortisol**	**Fat**	
**BMI**	B	-0.233	0.219								
	HERSELF	0.111	0.101								
	P	0.034	0.037								
**Professional activity**	B		0.255								
	HERSELF		0.094								
	P		0.007								
**Lactation period**	B	-0.285		0.575						0.615	
	HERSELF	0.121		0.098						0.098	
	P	0.020		0.001						0.001	
**HBD**	B							-0.242	-0.281		
	HERSELF							0.119	0.122		
	P							0.046	0.024		
**GROUP 6K**		**DPPH**	**Carbohyd rates**		**GROUP 6L**	**DPPH**	**Toto protein**	**Carbohydrates**	**Energy value**	**GROUP 6M**	**DPPH**
**HBD**	B	-0.311									
	HERSELF	0.113									
	P	0.008									
**Type of birth**	B	0.240							0.244		
	HERSELF	0.109							0.119		
	P	0.031							0.044		
**Professional activity**	B		0.251								
	HERSELF		0.117								
	P		0.035								
**Disease**	B	0.396									
	HERSELF	0.122									
	P	0.001									
**Age**	B						-0.425	-0.379			
	HERSELF						0.133	0.132			
	P						0.022	0.006			
**BMI**	B					0.349					

	HERSELF	0.125	
	P	0.007	
**Residence**	B		0.356
	HERSELF		0.158
	P		0.031

BMI – body mass index; FRAP – iron ion reduction capacity [μM]; DPPH – 2.2-diphenyl-1-picrylhydrazyl radical [% inhibition]; cortisol – concentration in ng/ml; fat. total protein. carbohydrates. dry matter – content in human milk in g/100ml; energy value – content in kcal/100ml; b – regression coefficient; SE – standard error from the regression coefficient. p – statistical significance level < 0.050
